# Longitudinal peak strain detects a smaller risk area than visual assessment of wall motion in acute myocardial infarction

**DOI:** 10.1186/1476-7120-8-2

**Published:** 2010-01-11

**Authors:** Lene Rosendahl, Peter Blomstrand, Lars Brudin, Tim Tödt, Jan E Engvall

**Affiliations:** 1 Department of Clinical Physiology, Ryhov County Hospital, Jönköping, Sweden; 2 Center for Medical Image Science and Visualization, CMIV, Linköping University Hospital, Linköping, Sweden; 3 Department of Clinical Physiology, Kalmar County Hospital, Kalmar, Sweden; 4 Department of Medical and Health Sciences, Linköping University Hospital, Linköping, Sweden; 5 Department of Medical and Health Sciences/CVM/Division of Cardiology, Linköping University Hospital, Linköping, Sweden; 6 Department of Medical and Health Sciences/CVM/Division of Clinical Physiology, Linköping University Hospital, Linköping, Sweden

## Abstract

**Background:**

Opening of an occluded infarct related artery reduces infarct size and improves survival in acute ST-elevation myocardial infarction (STEMI). In this study we performed tissue Doppler analysis (peak strain, displacement, mitral annular movement (MAM)) and compared with visual assessment for the study of the correlation of measurements of global, regional and segmental function with final infarct size and transmurality. In addition, myocardial risk area was determined and a prediction sought for the development of infarct transmurality ≥50%.

**Methods:**

Twenty six patients with STEMI submitted for primary percutaneous coronary intervention (PCI) were examined with echocardiography on the catheterization table. Four to eight weeks later repeat echocardiography was performed for reassessment of function and magnetic resonance imaging for the determination of final infarct size and transmurality.

**Results:**

On a global level, wall motion score index (WMSI), ejection fraction (EF), strain, and displacement all showed significant differences (p ≤ 0.001, p ≤ 0.001, p ≤ 0.001 and p = 0.03) between the two study visits, but MAM did not (p = 0.17). On all levels (global, regional and segmental) and both pre- and post PCI, WMSI showed a higher correlation with scar transmurality compared to strain. We found that both strain and WMSI predicted the development of scar transmurality ≥50%, but strain added no significant information to that obtained with WMSI in a logistic regression analysis.

**Conclusions:**

In patients with acute STEMI, WMSI, EF, strain, and displacement showed significant changes between the pre- and post PCI exam. In a ROC-analysis, strain had 64% sensitivity at 80% specificity and WMSI around 90% sensitivity at 80% specificity for the detection of scar with transmurality ≥50% at follow-up.

## Background

The treatment of acute myocardial infarction has undergone dramatic changes in the last decade. For ST-elevation myocardial infarction (STEMI), mechanical opening of the infarct related artery has gained widespread acceptance and the health care systems in many countries have adopted this policy for proper care of STEMI. Several studies have shown that short time to primary percutaneous coronary intervention (PCI) in patients with myocardial infarction reduces mortality [1-4], is associated with a high degree of myocardial salvage [5] and improves the procedural success rate of PCI, the functional recovery of the left ventricle and the clinical outcome [6]. Myocardium at risk, collateral flow, and duration of coronary occlusion are each independently associated with final infarct size [7] and the myocardial salvage achieved by reperfusion therapy in patients with acute myocardial infarction has a prognostic value for clinical outcome [8].

Research has tried to elucidate the relative importance of various time delays [3,9-11], different ways to protect ischemic myocardium as well as to find methods to predict the chance for success in infarct limiting therapies [12]. Such methods have relied on echo wall motion, echo measurements of deformation, scintigraphic signs of preserved myocardial blood flow as well as newer imaging methods such as gadolinium based visualization of micro vascular obstruction or oedema sensitive imaging of myocardial area at risk.

Echo wall motion analysis of myocardial ischemia is built on the concept that ischemia and scar confer a reduction in wall thickening and in longitudinal wall displacement and induce a delay in the onset of myocardial contraction. Various methods have been suggested for objective measurement of wall motion abnormalities [13,14] and experiments have been designed to measure the smallest temporal changes that the human eye can detect [15]. Strain (ε) expresses the local deformation of contracting muscle [16-18]. It is a complicated measure that requires 9 tensor values to adequately describe motion in all directions. Simplified solutions are those that determine strain along the tissue Doppler beam (1-dimensional) or from speckle in the gray scale image (2D-strain, 2-dimensional). 2D or 3D strain can also be calculated from tag lines introduced in cardiac tissue at a cardiac magnetic resonance imaging (MRI) exam. Strain appears to be less affected by global cardiac motion and the tethering effect of adjacent myocardial segments than myocardial velocities [19]. Normal ε values for a group of healthy adults have been defined [20]. Strain has been shown to quantify the severity of myocardial segmental dysfunction [21,22] as well as predict the recovery of regional wall motion in patients with acute myocardial infarction subjected to PCI [23]. For patients with acute myocardial ischemia, an ultrasonic strain index ((ε peak - ε systole)/ε peak) has been suggested for the differentiation of acutely ischemic segments from both normal and chronically dysfunctional myocardium [24]. However, despite being less sensitive to influences from neighbouring segments, the wide variation in reference values [20] has seriously hampered the use of these measurements for individual prediction in clinical practice.

Late gadolinium enhancement (LGE) MRI accurately determines infarct size [25] and has a high reproducibility [26]. A high spatial resolution enables measuring infarct transmurality and from this parameter the assessment of viable myocardium is possible [27-29].

In this study, we performed echocardiography with tissue Doppler imaging simultaneously with primary PCI, on the catheterization table, to determine myocardial area at risk and to study whether wall motion score index (WMSI) and tissue Doppler parameters correlated globally, regionally and segmentally with the size of the final scar and its transmurality.

## Methods

### Study Population

Twenty-six patients (23 men) average age 65 ± 8 years (range 50 - 78), table 1, were selected for this analysis from among 99 patients included in a study of primary PCI for ST-elevation myocardial infarction, from February 2006 to August 2007. The patients agreed to have acute echocardiography performed immediately as they were prepared for acute coronary intervention and on their return 4-8 weeks later for the determination of infarct size with MRI. Exclusion criteria were a new myocardial infarction (MI) and the need for rapid coronary artery bypass graft (CABG) surgery or renewed PCI during the interim period between primary discharge and the MRI. In the main study 159 patients were included and 99 finally completed the investigations. Acute echocardiography was possible only during office hours, enabling the inclusion of 26 patients for the echo substudy. All patients underwent coronary angiography with balloon dilatation and most frequently stenting, via a standard femoral approach. The culprit lesion was located in the left anterior descending coronary artery system (LAD) in 15 patients, in the right coronary artery (RCA) in 9 and in the left circumflex artery (CX) in 2 patients, table 1. Additional stenoses not dilated at the index event were seen in 10 of the 26 patients.

**Table 1:** Patient characteristics

	Parameter
Age (Yrs; mean (SD) range)	65.3 (7.9) 50-78
Gender	
Males (n; %)	23 (88)
Females (n; %)	3 (12)
	
Height (cm; mean (SD) range)	178 (9) 157-195
Weight (kg; mean (SD) range)	83 (9) 58-102
BMI (mean (SD) range)	26.2 (3.3) 21-37
Prior myocardial infarction	
Yes (n; %)	3 (12)
No (n; %)	23 (88)
Culprit coronary artery (angio)	
LAD (n; %)	15 (58)
RCA (n; %)	9 (35)
CX (n; %)	2 (8)
MI (extent)	
No MI	2 (8)
Subendocardial (n; %)	9 (35)
Transmural (n; %)	15 (58)

BMI = body mass index, LAD = left anterior descending coronary artery, RCA = right coronary artery, CX = left circumflex coronary artery, MI = myocardial infarction

Approval was obtained from the Regional ethical review board in Linköping. The study complied with the Declaration of Helsinki and with agreements on Good Clinical Practice. All patients gave written informed consent.

### Echocardiography

At least three apical views were obtained with tissue Doppler (two-beat loops) while the patient was draped and prepared for acute PCI (GE V7 or GE V5 with 3 MHz transducers using harmonic imaging technology). At follow-up, a minimum of three apical loops were recorded, preferably in end expiration to minimize translational movement of the heart. The left ventricle was divided into 16 segments (6 basal, 6 mid, and 4 apical) [30]. The myocardial motion of each segment was evaluated according to the standard American Society of Echocardiography wall motion scoring system and WMSI was calculated [30,31] (additional file 1). The three observers were blinded as to the temporal order of the echocardiographic investigations as well as to the result of the determination of transmurality.

**Figure 1:**
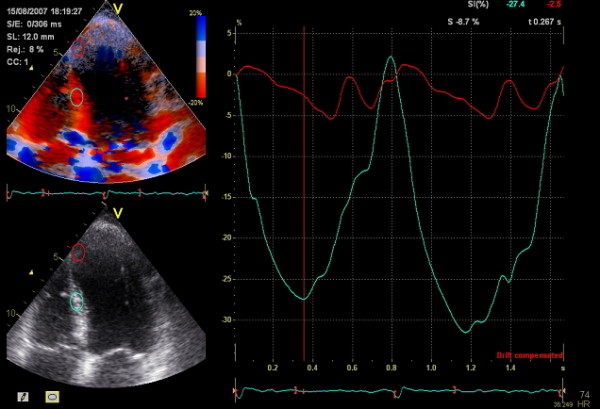
**Strain curves from the septum at follow-up**. Blue: normal longitudinal strain curve recorded from healthy myocardium in the middle septal segment. Red: reduced longitudinal strain in thinned, infarcted myocardium of the apical septal segment.

Tissue Doppler images were acquired with a frame-rate exceeding 90/s. Off-line analysis was performed using Echopac software (Echopac BT08, GE Vingmed Ultrasound, Norway). In each of the three apical views, six segments were defined and regions of interest (ROI) using sample volumes of 6 × 12 mm were applied. Tissue Doppler values in the apical anteroseptal and inferoseptal segments were averaged into an apical septal segment and in the anterolateral and inferolateral averaged into an apical lateral segment thus allowing conversion from 18 segments into a 16 segment model. After checking for aliasing in the velocity mode, myocardial strain and displacement curves were drawn and the peak values, regardless of delay time, were measured on two consecutive beats, if possible (figure 1, additional file 2). Peak systolic strain was calculated from the velocity determination in the longitudinal direction. Normal shortening strain was denoted with negative values while lengthening was assigned positive values. Displacement from the base towards the apex was given negative values and displacement towards the base positive values [32] (additional file 3). Long axis left ventricular function was assessed from mitral annular motion (MAM) which was measured in four positions on the mitral annular ring. Values for strain, displacement and MAM were obtained by two observers and their average was calculated. The reference values for longitudinal strain by Kowalski et.al. (-16% ± 5%) were used as cut-off [20]. Left ventricular ejection fraction was calculated using the biplane Simpson's method of discs and averaged from two consecutive heart beats in exams with sufficient image quality.

To obtain global measurements of wall motion and strain, measurements from each segment were added and the sum divided by the total numbers of segments. To assess regional measurements of wall motion and strain in relation to infarct transmurality, numbers for the three segments of each wall (anterior, lateral, posterior and septal) were averaged.

### Magnetic resonance imaging

The patients were placed in the magnet (1.5 T Achieva, Philips Healthcare, Best, The Netherlands) in supine position. A circular polarized body-array surface coil was used in all measurements. ECG-triggered MR images were obtained during repeated breath-holds.

Cine-MR imaging was performed with a balanced steady state free precession turbo field-echo (b-SSFP TFE) sequence that covered the entire left ventricle with on average 18 (range 10 - 23) short-axis slices and three long axis planes (apical 2-, 3- and 4-chamber views). Temporal resolution ranged between 26 - 41 ms (30 acquired phases). The inversion recovery turbo field echo (IR-TFE) sequence was a segmented 3D spoiled gradient echo sequence with TE = 1.3 ms, TR = 4.4 ms and TFE factor 43, leading to an acquisition phase time of 188 ms acquired during diastole. Slice thickness was 10 mm, intersection gap -5 mm (i.e. slices were overcontiguous), field-of-view 350 mm and image matrix 128 × 256. The contrast-enhanced images were acquired at the same slice positions as the cine-images, about 20 min after the administration of gadopentetate dimeglumine (Gd-DTPA) 0.2 mmol/kg bodyweight (Schering Nordiska AB, Järfälla, Sweden). Optimal contrast between hyperenhanced areas and normal myocardium was maintained by continually adjusting the inversion time to null the signal from the healthy myocardium.

**Figure 2:**
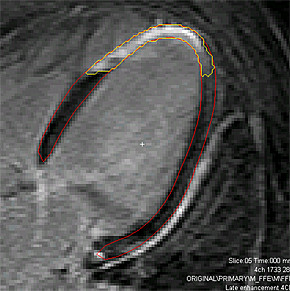
**Segmentation of the left ventricle with determination of transmurality**. Four-chamber view of the left ventricle. Red denotes the segmentation of the myocardium, yellow the scar, determined with "Segment". Transmurality is expressed as scar percentage of the area of the segment.

Scar size was measured by two different observers on short-axis images using a freely available software "Segment" http://segment.heiberg.se, [33]. Infarct volume and percentage was calculated from the short axis stack of slices (scar area × thickness × number of slices) and was averaged for the two observers. Infarct transmurality per segment was in this setting determined in the three apical views by using "Segment" and defined as segmental scar area (6 segments per view, figure 2). The 18 segments were reduced to 16 segments, identical to those used in the tissue Doppler analysis. Enddiastolic myocardial and cavity volumes were measured from the short axis LGE images (segmented myocardial or cavity area × thickness × number of slices). Myocardial mass (gram) was obtained by multiplying volume (ml) by 1.05

### Data analysis and statistics

All heart-related parameters were reasonably well normally distributed and presented as mean ± (SD). The difference on global level between pre- and post PCI exams was analysed by two-sided t-test for paired observations. Spearman's rank correlation was used for global-, regional-, and segmental functional parameters vs. infarct size and infarct transmurality. The difference between normal segments (transmurality < 1%) and segments with transmurality ≥ 50% on regional level was analyzed with Mann-Whitney's U-test. Receiver-operator-characteristics (ROC) curve analyses were performed using the statistical software MedCalc^® ^Version 6.10 (MedCalc Software, Mariakerke, Belgium). The interaction between WMSI and strain on the detection of segments with a transmurality ≥50% was analysed with logistic regression.

Interobserver variability of the functional measures was expressed as standard error of a single determination (S_method_) using the formula, first proposed by Dahlberg [34], S_method _= √(∑d_i_^2^/(2n)), where d_i _is the difference between the i:th paired measurement and n is the number of differences. S_method _was also expressed as % over all means when applicable.

Analyses were performed using SPSS 13.0 (SPSS Inc, Chicago, Illinois). Two-tailed *P *values were used, with p ≤ 0.05 considered to indicate statistical significance.

## Results

### Global left ventricular measures

Left ventricular myocardial volume was 167.8 ± 36.9 ml (range 88 - 254 ml). Infarct size, determined at follow-up 4 - 8 weeks after PCI, was on average (± SD) 14.9 ± 5.6 ml (8.7 ± 7.4% of the volume of the left ventricular myocardium). Mean transmurality of affected segments was calculated for each of the 24 patients with follow-up scars ≥ 1%, giving a patient average of 44.3 ± 18.0% (range 8.5 - 76). At follow-up, WMSI improved from an average (± SD) of 1.6 ± 0.3 to 1.4 ± 0.3 (n = 26; p = 0.001).

Corresponding numbers for ejection fraction were 38.5 ± 8.5% to 46.8 ± 8.5% (n = 17; p = 0.001) and for global strain -13.2 ± 3.3% to -15.7 ± 3.5% (n = 26; p < 0.001). Displacement changed from -5.4 ± 1.8 mm to -6.1 ± 1.4 mm (n = 26; p = 0.030). The change in MAM did not reach statistical significance (mean 10.5 ± 2.7 mm to 11.1 ± 2.3 mm; n = 26; p = 0.167).

**Figure 3:**
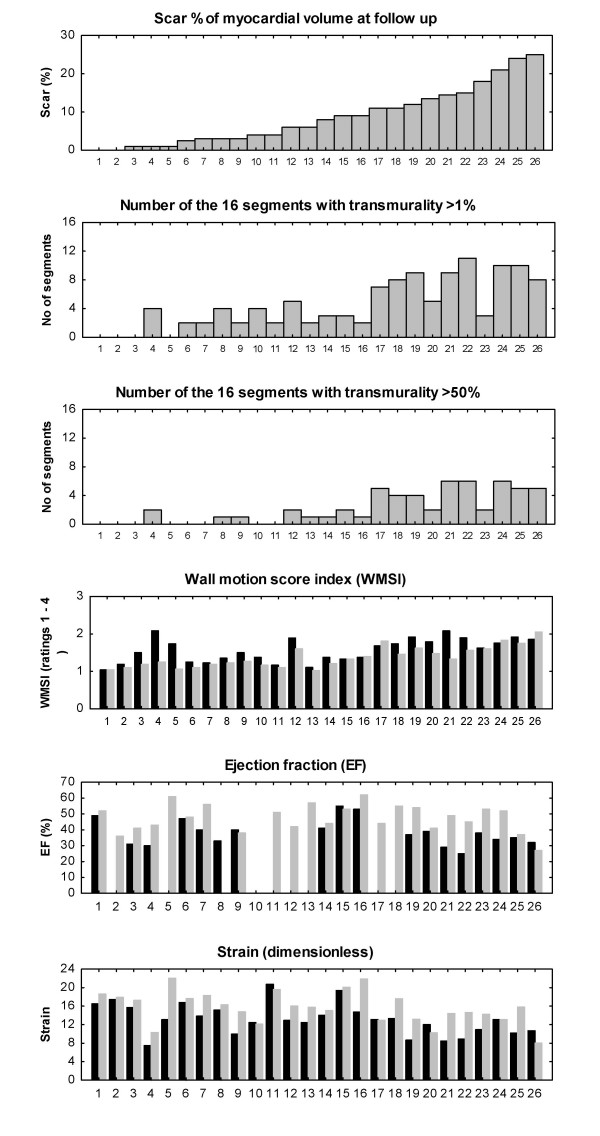
**Composite display of infarct size and functional measures**. Upper panel shows the distribution of scar percentage among the individual patients (no 1 to no 26). Next two panels show the number of segments with transmurality either >1% or >50% per patient. The three panels at the bottom show wall motion, ejection fraction and strain pre-PCI (black) and at follow-up (gray).

There were statistically significant correlations between infarct size and percent transmurality post-PCI (assessed by MRI) on the one hand and systolic ultrasonic measures pre and post PCI on the other (table 2). The highest correlation was for WMSI post PCI vs. infarct size (r = 0.83) and for WMSI post PCI and transmurality (r = 0.88), but also global strain post PCI correlated with infarct size (r = 0.51) and with transmurality (r = 0.64). Individual functional global measures pre- and post-PCI, ranked after scar size post-PCI, are shown in figure 3.

**Table 2:** Spearman rank correlation of global functional parameters vs. infarct size and infarct transmurality

**Parameter**	**Infarct size**	**Transmurality (%)**	
		
	**Scar%**	**All segments**	**Affected segments**
Strain (ε)			
Pre_PCI	**n = 26; r = 0.48; p = 0.014**	**n = 26; r = 0.61; p = 0.001**	n = 22; r = 0.42; p = 0.054
Post-PCI	**n = 26; r = 0.51; p = 0.008**	**n = 26; r = 0.64; p = < 0.001**	**n = 22; r = 0.44; p = 0.042**
MAM			
Pre_PCI	n = 26; r = -0.24; p = 0.245	n = 26; r = -0.39; p = 0.051	n = 22; r = -0.29; p = 0.198
Post-PCI	**n = 26; r = -0.56; p = 0.003**	**n = 26; r = -0.61; p = < 0.001**	n = 22; r = -0.40; p = 0.066
EF%			
Pre_PCI	n = 18; r = -0.29; p = 0.238	**n = 18; r = -0.52; p = 0.029**	n = 16; r = -0.40; p = 0.121
Post-PCI	n = 24; r = -0.06; p = 0.790	n = 24; r = -0.18; p = 0.411	n = 20; r = -0.07; p = 0.757
WMSI			
Pre_PCI	**n = 26; r = 0.55; p = 0.003**	**n = 26; r = 0.75; p = < 0.001**	**n = 22; r = 0.59; p = 0.004**
Post-PCI	**n = 26; r = 0.83; p = < 0.001**	**n = 26; r = 0.88; p = < 0.001**	**n = 22; r = 0.80; p = < 0.001**

Spearman's rank correlations (n; r; p) between global ultrasonic systolic measures pre and post PCI on the one hand and infarct size and transmurality post PCI on the other. Significant correlations are bolded.

### Regional ventricular measures

Wall motion score correlated moderately with transmurality in the acute phase and at follow-up (r = 0.67, p < 0.001 and r = 0.63, p < 0.001) while the correlation for longitudinal strain vs. transmurality was lower at both time points (r = 0.51, p < 0.001 and r = 0.44 p < 0.001) and for MAM still lower (r = -0.25, p = 0.01 and r = -0.41, p < 0.001). In a comparison between walls with transmurality ≥ 50% and normal walls, WMSI, strain and MAM all displayed values that were significant. The change post-pre was not significant for any of the three measures, see table 3.

**Table 3:** Regional analysis of wall motion score index

		Transmurality post-PCI				
			
		Normal (<1%)	1-24%	25-50%	>50%	p-value*
**n = number of walls**		50	28	11	15	
**Transmurality (%; mean)**						
Postop	n = 104	0.0 ± 0.1	9.4 ± 6.5	36.3 ± 7.4	64.7 ± 9.9	-
**WMSI (mean ± SD)**						
Preop	n = 102	1.3 ± 0.4	1.7 ± 0.3	1.9 ± 0.2	2.2 ± 0.5	p < 0.001
Postop	n = 104	1.2 ± 0.3	1.4 ± 0.4	1.6 ± 0.3	1.9 ± 0.2	p < 0.001
Δ	n = 102	-0.1 ± 0.4	-0.3 ± 0.4	-0.3 ± 0.4	-0.3 ± 0.6	0.330
**Strain (mean ± SD)**						
Preop	n = 102	-15.3 ± 4.9	-10.5 ± 3.6	-10.9 ± 4.5	-8.4 ± 6.3	p < 0.001
Postop	n = 104	-17.3 ± 4.7	-14.3 ± 4.2	-14.9 ± 4.1	-12.3 ± 4.2	p < 0.001
Δ	n = 102	-2.0 ± 5.8	-4.2 ± 4.6	-4.0 ± 3.7	-4.0 ± 5.0	0.205
**MAM (mm; mean ± SD)**						
Preop	n = 98	11.4 ± 2.9	9.4 ± 2.9	10.7 ± 3.0	9.3 ± 2.9	0.027
Postop	n = 103	12.2 ± 2.6	10.1 ± 2.1	11.6 ± 2.1	9.2 ± 2.0	p < 0.001
Δ	n = 97	0.8 ± 2.9	0.6 ± 2.8	1.4 ± 3.0	0.2 ± 2.2	0.706

Regional (septal, anterior, lateral and inferior wall) analysis of wall motion score index (WMSI), strain (ε) and mitral annulus movement pre and post PCI calculated for the different outcomes of postoperative transmurality.

Footnotes: *; significance levels for the difference between normal (<1%) and >50% transmurality

### Segmental ventricular measures

In the initial study, 390 out of 416 segments (94%) were successfully visualised and at follow up 410 of the 416 segments (99%). Wall motion score correlated moderately with transmurality in the acute phase and at follow-up (r = 0.58, p < 0.01 and r = 0.53, p < 0.01) while longitudinal strain correlated weakly with transmurality at both time points (r = 0.38, p < 0.01 and r = 0.31 p < 0.01). Displacement, regardless of the position of the segment (apical-middle-basal) also correlated weakly with transmurality (r = 0.28, p < 0.001 and r = 0.34, p < 0.001).

The segmental values were analysed from two approaches. One was based on the evaluation of WMSI (normal or abnormal) and transmurality in regard to strain for segments that either deteriorated, improved or remained unchanged between the acute event and follow-up (table 4). In the other approach, normal or reduced strain was the watershed from which the level of transmurality and changes in WMSI were evaluated (table 5).

**Table 4:** Segmental analysis of strain (ε) pre and post PCI and transmurality post-PCI

PrePCI WMSI		Normal		Abnormal		
**Follow-up WMSI**		**Deteriorated**	**Unchanged**	**Deteriorated**	**Unchanged**	**Improved**
**n**		7	152	15	141	75
**WMSI (mean ± SD)**						
Preop		1.0 ± 0.0	1.0 ± 0.0	1.6 ± 0.4	1.8 ± 0.6	2.3 ± 0.5
Postop		1.7 ± 0.2	1.1 ± 0.1	2.3 ± 0.4	1.6 ± 0.6	1.3 ± 0.5
Post-pre-difference		0.7 ± 0.2	0.1 ± 0.1	0.7 ± 0.2	-0.2 ± 0.2	-1.0 ± 0.3
**Strain (mean ± SD)**						
Preop		-15.1 ± 5.7	-16.6 ± 5.6	-11.8 ± 7.1	-11.4 ± 8.0	-9.4 ± 7.9
Postop		-16.6 ± 5.2	-17.4 ± 5.2	-13.4 ± 8.1	-15.0 ± 7.7	-14.3 ± 6.8
Post-pre-difference		-1.5 ± 5.1	-0.8 ± 6.5	-1.6 ± 3.7	-3.7 ± 7.3	-4.9 ± 8.3
**Strain (segments; n (%))**						
Preop						
Normal		5 (71)	129 (85)	9 (60)	86 (61)	32 (43)
Abnormal		2 (29)	23 (15)	6 (40)	55 (39)	43 (57)
Postop						
Deteriorated		1 (14)	25 (16)	0 (0)	11 (8)	9 (12)
Unchanged		4 (57)	89 (59)	11 (73)	79 (56)	31 (41)
Improved		2 (29)	38 (25)	4 (27)	51 (36)	35 (47)
**Transmurality (segments; n (%))**						
Postop						
Normal (<1%)		7 (100)	142 (93)	9 (60)	82 (58)	43 (57)
≥ 1%		0 (0)	10 (7)	6 (40)	59 (42)	32 (43)
> 50%		0 (0)	0 (0)	5 (33)	25 (18)	20 (27)

Segmental analysis of strain (ε) pre and post PCI and infarction transmurality post-PCI calculated for normal and abnormal PCI wall motion score index (WMSI) preoperatively, in turn related to the different outcomes postoperatively (deteriorated, unchanged and improved).

**Table 5:** Segmental analysis of wall motion score index (WMSI) pre and post PCI and transmurality post PCI

**PrePCI strain**		**Normal**			**Abnormal**		
	
**Follow-up strain**		**Deteriorated**	**Unchanged**	**Improved**	**Deteriorated**	**Unchanged**	**Improved**
**n**		44	162	55	2	52	75
**Strain (mean ± SD)**							
Preop		-21 ± 5	-17 ± 4	-15 ± 3	-1 ± 4	-6 ± 5	-4 ± 6
Postop		-12 ± 6	-17 ± 4	-24 ± 4	7 ± 6	-8 ± 5	-16 ± 6
Post-pre-difference		-6 ± 3	-6 ± 3	-7 ± 4	-1 ± 1	-3 ± 3	-4 ± 4
**WMSI (mean ± SD)**							
Preop		1.4 ± 0.5	1.3 ± 0.5	1.4 ± 0.4	2.6 ± 0.1	1.9 ± 0.7	2.1 ± 0.8
Postop		1.2 ± 0.3	1.3 ± 0.4	1.3 ± 0.4	2.6 ± 0.1	1.7 ± 0.7	1.6 ± 0.6
Post-pre-difference		1.2 ± 0.4	1.1 ± 0.4	1.0 ± 0.4	1.0 ± 0.0	1.1 ± 0.6	1.4 ± 0.5
**WMSI (segments; n (%))**							
Preop							
Normal		26 (59)	83 (51)	25 (45)	0 (0)	10 (19)	15 (20)
Abnormal		18 (41)	79 (49)	30 (55)	2 (100)	42 (81)	60 (80)
Postop							
Deteriorated		1 (2)	8 (5)	5 (9)	0 (0)	7 (13)	1 (1)
Unchanged		34 (77)	136 (84)	45 (82)	2 (100)	32 (62)	44 (59)
Improved		9 (20)	18 (11)	5 (9)	0 (0)	13 (25)	30 (40)
**Transmurality (segments; n (%))**							
Postop							
Normal (<1%)		40 (91)	130 (80)	44 (80)	1 (50)	27 (52)	41 (55)
≥ 1%		4 (9)	32 (20)	11 (20)	1 (50)	25 (48)	34 (45)
> 50%		1 (2)	11 (7)	3 (5)	1 (50)	16 (31)	18 (24)

Segmental analysis of wall motion score index (WMSI) pre and post PCI and infarction transmurality post PCI calculated for the different outcomes of strain (ε) pre-PCI, in turn related to the different outcomes postoperatively (deteriorated, unchanged and improved).

### Myocardial area at risk

**Figure 4:**
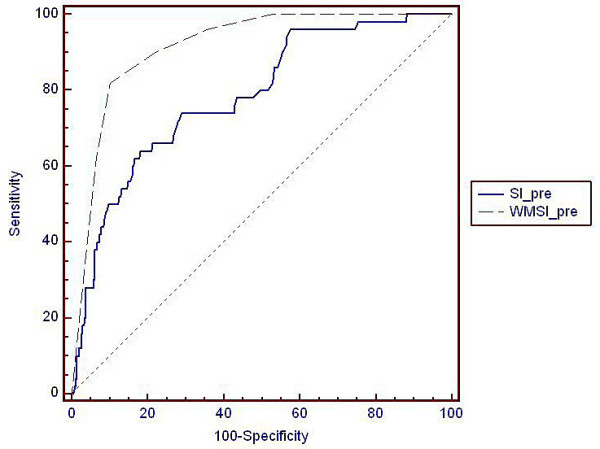
**ROC curves for WMSI and strain vs transmurality ≥50%**. ROC-curves displaying the interrelationship between sensitivity and specificity for wall motion score index and strain vs. the detection of segments with a transmurality ≥50%. Area-under-curve for WMSI is 0.92 and for strain 0.78, p < 0.0001. WMSI = Wall motion score index, SI = peak longitudinal strain

Initially, 231 out of 390 visible segments displayed wall motion abnormalities. 97 of these developed scar with transmurality > 1%, i.e. final scar affected 42% of the segments at risk (table 4). Based on longitudinal strain > -11%, 129 segments were abnormal initially of which 60 developed scar with transmurality > 1%, i.e. final scar affected 46% of the segments at risk (table 5). However, of the 261 segments that were determined to be normal according to strain < -11%, 47 segments (18%) developed scar compared to 10 segments (6%) with WMSI. The two methods were compared in a ROC-analysis as for the prediction of segments that were to develop scar with a transmurality ≥ 50%, figure 4. AUC was significantly higher for WMSI (0.92) than for strain (0.78), p < 0.0001. Sensitivity at 80% specificity was for strain 64% and for WMSI close to 90%. In a logistic regression analysis incorporating WMSI and strain, both parameters were significant for the prediction of transmurality ≥ 50% but strain did not add significant information beyond that carried by WMSI.

### Interobserver variability

The calculated error (S_method_) between three observers were for WMSI 0.50 (coefficient of variation (COV) = 32%) and 0.42 (COV = 30%) calculated for the pre- and post-PCI investigations. Corresponding methodological errors for the two observers of the MAM were 1.30 mm (12.4%) and 1.04 (9.3%), respectively. The methodological error in absolute numbers was for strain pre-PCI 5.4 and for post-PCI 4.5. Corresponding values for displacement were 1.59 and 1.43, respectively. COV for these measurements is of no interest since the values include zero. No significant differences were found, in regard to these measurements, between pre- and post PCI examinations. S_method _for scar assessed by MR post-PCI was 1.6 ml (11%) or related to myocardial volume 1.1% (12%).

## Discussion

### Main findings of the study

In this study we show that the initial evaluation based on visual assessment of wall motion as well as on objective measurements of wall deformation correlate with the final infarct size and transmurality at follow-up after 4 - 8 weeks. Functional evaluation based on echocardiography on the catheterization table seems valid, since those measurements correspond fairly well with assessments performed under less hurried conditions at follow-up. Prediction of segments/walls that will develop permanent scar is possible with both methods but visual assessment seems superior in this respect (figure 4). Visual wall motion assessment has been criticized because of its subjectivity [35] but was robust in the present study. Peak strain from myocardial tissue Doppler showed less correlation with final infarct size and transmurality than visual assessment, both in the acute study and in the quiet imaging conditions at follow-up.

### Global measurements

Global strain reflects the averaged segmental myocardial long-axis relative shortening and is a global functional measurement that may give information beyond what is available from WMSI and left ventricular ejection fraction (LVEF) [36]. Strain is an objective measurement compared with the subjective evaluation of wall motion and might therefore be a valuable instrument in the daily workflow. However, we found that global strain showed a moderate correlation with total infarct size and mean transmurality, lower than that for WSMI. To the contrary, Gjesdal et.al. showed a higher correlation (0.84) between global strain and scar compared to WMSI and scar (0.70) in patients with chronic myocardial infarction [37]. In their study, 2D-speckle tracking echocardiography was used on somewhat younger patients (mean age 55 yrs) 9 months after MI. Additionally, Vartdal et. al found a correlation of 0.77 between strain and infarct size in patients with acute STEMI [38]. The corresponding figure for WMSI and infarct size was r = 0.45. However, these patients were examined 1.5 h after revascularization when ischemic wall motion abnormalities could have declined, possibly faster than changes in strain. Interestingly, they also found that global strain might be a valuable predictor for the total amount of scar and hence might be a clinical tool for risk stratification. Recently, Gjesdal. et.al. showed a significant correlation between MAM, measured in 4 positions, and infarct size (r = 0.58, p < 0.01) in patients with chronic scar 9 months post AMI [39]. Additionally, Sjøli et.al. showed a correlation of 0.62 between global strain and infarct size measured within 3.5 h after revascularization [40]. Global strain showed a higher correlation with the size of myocardial scar compared with LVEF. This is in line with our results showing that LVEF has only a weak correlation with infarct size and mean transmurality (table 2). Ugander et.al. also confirmed a large variation in LVEF in relation to infarct size and concluded that infarct size cannot be used to predict LVEF [41]. The number of dysfunctional adjacent segments seemed to be a more important determinant on regional wall function than infarct transmurality [42]. This could be caused by extensive hibernation or a compensatory increase in wall motion in healthy parts of scarred ventricles.

### Regional measurements

By averaging measurements from the three segment levels in each wall, regional measurements could be correlated with regional scar. This was done to allow a comparison of strain and wall motion scoring with the regional motion amplitude of the mitral annulus, which could easily be obtained from tissue Doppler velocities integrated over time ("tissue tracking"). We found a rather low correlation for regional MAM vs. transmurality, higher for regional strain and the highest for WMSI vs. transmurality.

### Segmental measurements

Evaluation of wall motion is very important in the clinical setting but draw-backs are the subjectivity [35] and the extended learning curve for the physician. Strain has been proposed to identify acute ischemia [24] and to grade myocardial dysfunction [21,32]. In this study we hypothesized that strain could quantify left ventricular function in relationship to scar transmurality. However, we found a higher correlation between wall motion and scar transmurality compared with strain vs. transmurality, as well as displacement vs. transmurality, both pre- and post PCI. Despite the theoretical advantages of strain, visual assessment by three experienced observers performed better when predicting scar transmurality in both the acute and chronic settings. Displacement, correlated even more weakly than strain (r = 0.28 pre, r = 0.34 post). This is in line the finding of Skulstad et.al. who studied the relationship of strain and displacement for quantification of regional myocardial function [32].

### Myocardial area at risk

To determine myocardial salvage, an initial measure of myocardial area-at-risk is required. Myocardial oedema determined with MRI and myocardial perfusion determined with SPECT have been used, mainly because imaging can be delayed for several days for MRI and for 6-8 hours in regard to SPECT. In the present study, we selected wall motion assessment, either visual or by tissue Doppler, because of its low cost, availability at bedside, and the possibility to perform assessment in immediate relation to the intervention. We found that both methods identified segments that were to develop scar transmurality in excess of 50%. However, wall motion scoring performed better than strain measurement both in terms of detecting initially threatened segments and in avoiding false positive results. In total, 42% of threatened segments according to wall motion scoring developed significant myocardial scar. This result is in line with guidelines suggesting that the goal of acute infarct treatment is a final scar size < 40% of the initial risk area [12].

### Limitations

The first echocardiographic examination was performed under difficult scanning conditions, while preparing the patient for acute PCI resulting in a compromised image quality. However, correlations were in the same range as for measurements performed at follow-up, when the imaging conditions were favourable. Strain measurements are known to be load dependent [19,43], which can have influenced measurements performed under the acutely unstable hemodynamic conditions of myocardial infarction. While velocity, displacement and strain obtained with tissue Doppler are angle-dependent, 2D-speckle tracking adds a second dimension to the scanning plane which reduces the influence of off-angle effects. Accordingly, speckle tracking MRI has shown higher values for peak longitudinal strain of healthy segments (about -20%) vs our tissue Doppler values (-17) [44]. In the present study, speckle tracking was not performed because the underlying gray scale frame rate in the colour tissue Doppler loops was too low to allow good quality tracking Tissue Doppler produces a strong signal from the myocardial wall also in difficult imaging conditions and was thus projected to produce a more stable result than visual assessment. But, in the post processing analysis, strain curves turned out to be sensitive to the placement of the ROI's as well as to shadowing from the lungs.

In this study we have used a 16 segment model of the left ventricle in favour of the newer 17 segment model proposed by AHA [45]. However, since the 16 segments are identical with those of the AHA model and measurements were not performed on the apical cap, the model was not considered to have an influence on the result.

Registration (= aligning images from different studies) is a major problem when studies are performed on patients at different time points and with different modalities. However, WMSI, strain, displacement and infarct transmurality were all assessed in the long axis view and care was taken to align the imaging plane with that used for measuring transmurality.

## Conclusions

Echocardiography, acutely performed while preparing patients for primary PCI, detected myocardial area at risk using two methods: subjective assessment of wall motion as well as objective measurement of deformation (strain). These measurements, repeated at follow-up 4-8 weeks later, correlated with the development of scar transmurality in excess of 50%, which is considered to be the limit for the return of useful wall motion post intervention. Analysis based on receiver-operator-characteristics curves showed WMSI to be superior to peak longitudinal strain in this prediction. Thus, advanced technological analysis of wall motion did not contribute additional value compared with a careful visual assessment by an experienced observer.

## Competing interests

The authors declare that they have no competing interests.

## Authors' contributions

LR performed a major part of all measurements and in writing the manuscript. PB participated in evaluating some of the patients and reviewed the manuscript. LB participated in the statistical analysis of the results and in writing of the manuscript. TT participated in planning the study, was responsible for the angiographic evaluation and reviewed the manuscript. JE planned the study, investigated some of the patients, performed measurements and analyses and took a major part in writing the manuscript. All authors have read and approved the final manuscript.

## Funding

This project was supported by Futurum - the academy for healthcare, Jönköping County Council, the Swedish Heart Lung Foundation, the Swedish Research Council, the Medical Research Council of Southeast Sweden, the Centre for Medical Image Science and Visualization, Linköping University Hospital, and the faculty of Linköping University.

## Supplementary Material

Additional file 1 **Gray scale 4-chamber view**. Patient at follow-up, with thinning and akinesia in the distal part of the septum and in the apex. Click here for file

Additional file 2 **Doppler strain imaging 4-chamber view**. The same patient as above. The blue trace displays low and delayed peak longitudinal strain (5%) from the apical septal segment while the yellow trace from the normal middle segment of the septum displays normal peak longitudinal strain at about 30%. Click here for file

Additional file 3 **Tissue tracking 4-chamber view**. The same patient as above. Tissue tracking of the mitral annular excursion is somewhat low at 8.5 mm in both the septal and the lateral wall. It is not reduced in the septum despite the apical myocardial infarct. Click here for file
